# Urbanization and Disease Emergence: Dynamics at the Wildlife–Livestock–Human Interface

**DOI:** 10.1016/j.tree.2016.09.012

**Published:** 2017-01

**Authors:** James M. Hassell, Michael Begon, Melissa J. Ward, Eric M. Fèvre

**Affiliations:** 1 Institute of Infection and Global Health, The University of Liverpool, Leahurst Campus, Chester High Road, Neston, CH64 7TE, UK; 2 International Livestock Research Institute, Nairobi, Kenya; 3 Institute of Integrative Biology, The University of Liverpool, Liverpool L69 7ZB, UK; 4 Centre for Immunity, Infection and Evolution, University of Edinburgh, Edinburgh, UK

**Keywords:** disease emergence, wildlife, urbanization, interface

## Abstract

Urbanization is characterized by rapid intensification of agriculture, socioeconomic change, and ecological fragmentation, which can have profound impacts on the epidemiology of infectious disease. Here, we review current scientific evidence for the drivers and epidemiology of emerging wildlife-borne zoonoses in urban landscapes, where anthropogenic pressures can create diverse wildlife–livestock–human interfaces. We argue that these interfaces represent a critical point for cross-species transmission and emergence of pathogens into new host populations, and thus understanding their form and function is necessary to identify suitable interventions to mitigate the risk of disease emergence. To achieve this, interfaces must be studied as complex, multihost communities whose structure and form are dictated by both ecological and anthropological factors.

## Trends

Urbanization can create diverse wildlife–livestock–human interfaces.

Interfaces represent a critical point for cross-species transmission and emergence of pathogens.

Interfaces should be studied as complex, multihost communities.

Molecular epidemiology can add real-world complexity to the study of disease emergence.

## Emerging Diseases in Changing Landscapes

**Emerging infectious diseases** (**EIDs**) (see Glossary) are recognized as pathogens ‘whose incidence in host populations has increased within the past two decades or threatens to increase in the near future’ [1]. As well as describing the spread of newly evolved or previously undetected pathogens, pathogens that are increasing their geographic spread, increasing their impact, changing their clinical presentation or moving into human hosts for the first time, the term emergence can also be used to describe the reappearance (or re-emergence) of a known infection after a decline in incidence [1]. It is estimated that between 60 and 80% of newly emerging infections are zoonotic in origin and thus are (at least initially) dependent on an animal **reservoir** for survival 2, 3. Of these emerging zoonoses, at least 70% have a wildlife origin, with cross-species spread and onward transmission representing a natural response to the evolutionary pressures of pathogen ecology 3, 4. Although both wildlife and domesticated animal reservoirs can be considered important sources of EIDs, it is the anthropogenic influence on ecological systems that dictates the level of risk that operates at the **interface** between humans and animals in zoonotic disease emergence.

The impact of humans on the ecosystems within which they exist have occurred for as long as there have been humans. However, over the past 10 000 years, human–ecosystem interactions have become increasingly profound following a series of chronological transitions: (i) the establishment of local settlements, agriculture, and domestication of livestock; (ii) regional contact through trade; (iii) intercontinental exploration, imperialism, and industrialization; and (iv) globalization, urbanization, and climate change [5]. Current levels of human–ecosystem interaction, driven by increased environmental encroachment and **land-use change** (exploitation of natural resources and agricultural practices), and environmental effects such as climate change, will result in habitat alteration and changes in species assemblage and contact rates that promote the emergence of zoonotic disease. Spread and persistence of newly emerged (or re-emerged) pathogens can then be perpetuated by a combination of factors including expanding global human populations and urbanization, international trade and travel, intensive livestock keeping systems, proliferation of reservoir populations, and antimicrobial drug use 4, 6, 7, 8. Land-use change, through population-driven anthropogenic influences such as forestry, mining, agriculture, and urban and industrial development, is frequently associated with disease emergence 9, 10.

**Figure 1:**
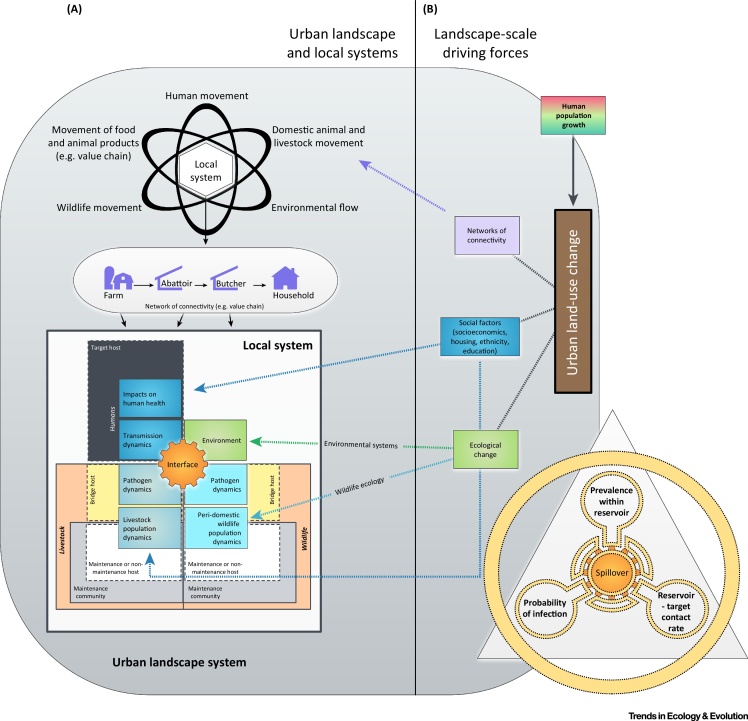
Conceptual Framework for Disease Emergence in Urban Landscapes (adapted, with permission, from 34, 38). It should be noted that we consider the structure of this framework as applicable to the emergence of antimicrobial resistance, as it is to disease emergence [92]. (A) This framework incorporates urban land-use change and its effects on two spatial scales: at a systems and local level. A simplified disease reservoir framework is included at the local level, in which livestock and synanthropic wildlife exist within the maintenance community as maintenance hosts (populations within the reservoir that can maintain the pathogen) or non-maintenance hosts (populations within the reservoir that cannot maintain the pathogen, therefore acting as vectors), or as bridge hosts that exist outside the maintenance community. (B) Following [38], spillover, which in this framework can relate to pathogen transfer in all directions except for target to reservoir, is governed by the force of infection consisting of the three elements shown.

Urbanization can be considered a key driver of land-use change that is likely to increase at an unprecedented rate in the coming decades, particularly in developing countries, where as much of 90% of population growth is projected to occur in cities 11, 12. Human population density and growth are significant predictors of historical EID events, and thus urbanization is likely to have a profound effect on public health as rural pathogens adapt to urban conditions, and other pathogens emerge (or re-emerge) in urban areas [3]. Human factors such as population density, migration, trade, sanitation, and access to clean water can promote the transmission of pathogens and alter vector dynamics, while social factors that drive health inequality (socioeconomic status, housing, race, ethnicity, gender, and education) also influence the epidemiology of infectious disease in urban areas 13, 14 (Figure 1). For cities in developing countries, the epidemiological effects of these factors are often concentrated in informal settlements, where population growth and density is highest [14]. In this review, we focus on rapid urbanization (predominantly a feature of developing countries) as a driver of disease emergence, and use it to explore how anthropogenic changes are driving interactions and the potential for disease emergence between sympatric wildlife, livestock, and humans.

## Urbanization and Disease Emergence

Spatial overlap between hosts, and overlap in vector ranges are key requirements for the emergence of directly transmitted and vector-borne pathogens, respectively. As such, in order to investigate the conditions in which urbanization might lead to the emergence of zoonotic disease across species, and thus risk factors for transmission to humans, it is necessary to simplify the complexity of urban systems by considering them as a network of interfaces across which pathogens can be transmitted; the physical interfaces at which humans and animals interact and pathogens are exchanged exist within the context of societal and policy interfaces (as depicted in the schematic in Figure 1). These networks exist at different scales. At a local-scale, households form part of what can be considered urban communities; groups of similar physical interfaces that are characterized by a set of societal (e.g., demographic and socioeconomic) characteristics. These communities are linked by the movement of people, livestock and their products, and wildlife, and the environment (which can conveniently be defined as networks of connectivity) 15, 16. As a result, key drivers that could promote interaction between humans and animals are: (i) livestock-keeping practices, production systems, and the movements of livestock and animal products in urban areas; and (ii) the direct effects of urbanization on the physical environment, ecosystems in which urban centers are developed, and animal communities that exist freely within these 8, 17. Urban systems are highly complex and the factors listed above are likely to influence the type and extent of human interactions with livestock, animal products, and ecosystems, resulting in the creating of human–animal interfaces that might promote the transmission of disease between animals and people.

Urban-adapted (referred to here as **synanthropic**) wildlife is abundant in cities, and is composed of species that can respond to behavioral and resource-based selection pressures imposed by urban environments [18]. Many synanthropic species have been shown to carry zoonotic pathogens and in some cases act as reservoir hosts for these pathogens. Studies generally focus on those species that are found ubiquitously within human environments and that commonly act as hosts for zoonotic diseases, such as rodents, birds, bats, and certain other species of mammal (e.g., foxes in Europe and raccoons in the US) 19, 20. Rodents, for example, harbor important zoonoses such as plague, leptospirosis, and hantavirus infection, and the emergence and re-emergence of these pathogens in human populations is seemingly linked to increasing urbanization and urban poverty in developing countries and the ecology of zoonotic pathogens in rat populations 17, 21, 22, 23. Anthropogenic changes associated with urbanization can also bring bats into closer contact with livestock and humans and alter disease ecology 24, 25. As such, human activities that increase exposure to populations of urban-dwelling wildlife species will undoubtedly increase the risk of pathogens spilling over to humans or livestock, but little is known of the epidemiological processes by which this occurs at such interfaces.

## Epidemiology at the Wildlife–Livestock–Human Interface

Most infectious agents circulate in communities composed of hosts that are infected with multiple parasites and parasites that can infect a variable diversity of hosts. Small changes in parasite community structure (within-host competition, or perturbations from host population dynamics) can result in far-reaching consequences for epidemiology of multihost and single host (monoxenous) parasite species 26, 27, 28. Such downstream epidemiological effects are demonstrated in several well-studied zoonotic disease systems, including the seasonal and co-infection dynamics of cowpox virus [29], Lyme disease in white-footed mice [30], and Nipah and Hendra virus in fruit bats 24, 31, 32. With the emergence of high-profile pathogens that exhibit wide host plasticity (such as Ebola and avian influenza viruses), a community approach is being increasingly embraced for studying the multihost ecology of zoonotic pathogens.

Studying the role of wildlife in multihost disease systems is complicated by ecological and behavioral attributes unique to these species, and the influence of natural and human systems; both of which complicate conceptual models of disease transmission [33]. Following the disease reservoirs framework recently revised by Viana *et al.* [34] and Caron *et al.* [35], in a multihost pathogen system where wildlife either exists within the **maintenance community** as a **maintenance host** or non-maintenance host, or outside the maintenance community as a **bridge host**, the dynamics of a zoonotic agent involve two phases: (i) transmission between maintenance and/or non-maintenance host species (wildlife and/or domestic) within the reservoir; and (ii) **spillover** transmission to humans from the maintenance community (Figure 1). In basic models, the persistence required for hosts to maintain a zoonotic pathogen and thus act as a maintenance community is determined by the **basic reproductive number** (**R**_**0**_: the transmission potential of a pathogen) and critical community size, while risk of spillover transmission to humans is defined by the force of infection from animals to humans. Contact is a key feature of both reservoir and disease emergence dynamics; R_0_ is closely linked to the rate of contact between susceptible and infectious individuals and the recovery or mortality rate of infected individuals, and the force of infection (and thus risk of human spillover) is determined by prevalence of infection in the maintenance population and/or bridge hosts, the rate of contact between humans and infected individuals, and the probability that infection occurs upon contact 36, 37, 38. However, host ecological traits (such as life-history characteristics, seasonality, coloniality, and sympatry) and population-level changes brought on by land-use change are likely to play a large role in pathogen transmission and persistence in wildlife and livestock species 33, 39. These factors (particularly human ecology) will strongly influence contact between wildlife, livestock, and humans, and prevalence of infection in animal reservoirs, and are therefore of fundamental importance to reservoir dynamics and disease emergence in changing landscapes.

Murray and Daszak [40] discuss two conceptual models for disease emergence under land-use change; the perturbation and pathogen pool hypotheses. The perturbation hypothesis focuses on a more dynamic model for disease emergence, where land-use change forces perturbations in pathogen dynamics within the reservoir, before emergence occurs in humans or livestock. The pathogen pool hypothesis assumes exposure to novel diseases from a diverse pool of pathogens in wildlife to which humans or livestock, as naïve hosts, have not had prior exposure. In reality, it seems unlikely that these two hypotheses are mutually exclusive; evidence from empirical studies generally favors a dynamic model for disease emergence [41]. As such, the extent to which perturbation (changes in species richness, abundance, and contact rate) or the zoonotic pathogen pool dictate risk of emergence at urban interfaces, is probably dependent on the impact of urbanization on community ecology, and the degree of coevolution between sympatric wildlife, humans, and livestock at each interface.

## Influence of Urbanization on Pathogen Dynamics within Multihost Wildlife Systems

Associations between urbanization and the prevalence of pathogens in populations of free-ranging wildlife have been described for a wide taxonomic range of host species and pathogens (reviewed in [13]). Evidence suggests that through altered habitat structure and changes to resource availability, urbanization results in significant changes to the structure of wildlife communities, which are subsequently characterized by low biodiversity with proportional increases in abundance of certain generalist species 42, 43. From a landscape-scale perspective, this results in a declining trend in species richness from rural areas to urban centers (biotic homogenization) with synanthropic species occurring at higher densities in urban and suburban environments than less-disturbed areas 13, 44. Not surprisingly, such profound changes in trophic structure will have epidemiological consequences for pathogens within these communities, and as a general rule, declining host biodiversity should be matched by a loss in parasite diversity, thus reducing the pathogen pool and with it the risk of novel disease emergence [45]. However, the epidemiological consequences of changes to such a system are likely to be pathogen specific, and dependent on how trophic reassortment affects the following parameters: likelihood of encounter and transmission between competent hosts, host abundance and/or density, and infected host mortality and recovery [46]. For example, helminth species richness of rodents in South East Asia is positively associated with decreasing rodent species richness, and increasing rodent abundance and level of synanthropy [47]. Increases in synanthropic species population density can elevate contact rates (through changes in host ranging patterns and densities), and thus increase the risk of pathogen transmission via direct contact and orofecal routes 37, 44. Fragmentation of these populations, in contrast, can result in genetic bottlenecks and subsequently reduced effective immune responses [48]. As host diversity decreases along gradients of urbanization, many pathogens are lost, but some (notably those in the hosts that remain in low diversity communities) can increase as a result of increased host abundance (termed the dilution effect) 30, 49. Reverse zoonotic transmission (zooanthroponosis) from humans to wildlife can also pose a threat to wildlife populations with increased exposure to humans 50, 51. The epidemiological effects of urbanization can therefore have important implications for both wildlife conservation and public health, with marginal wildlife species being susceptible to infection with pathogens circulating in urban-adapted hosts, and the potential for increased circulation of certain zoonotic pathogens in competent synanthropic reservoir hosts.

## Interfaces between Sympatric Wildlife, Livestock and Humans in an Urban Landscape

Wildlife populations in urban landscapes are heterogeneously distributed, and certain species group in spatial aggregations with livestock (or their products) and humans, creating interfaces that might be important for the transmission of zoonotic agents. As described, the dynamics of infection at these interfaces are determined by changes in diversity, abundance and contact rates between reservoir and **target hosts**, thus influencing risk of cross-species pathogen transmission. Several systematic reviews have identified high-risk interfaces for zoonotic disease transmission on a global scale; specific interfaces for spillover from wildlife include human dwellings, agricultural fields, and occupational exposure, while broader descriptions include agricultural intensification and environmental change 8, 52. However, as argued by Jones *et al.* [8], attempts to describe systems within which pathogens emerge or change in virulence have predominantly focused on global generalizations, which might not be appropriate to capture the heterogeneity of interfaces. Instead, interfaces and the driving factors that define them should be studied at appropriate, spatially explicit scales [53]. We consider these feedback loops at hypothetical urban wildlife–livestock–human interfaces in Box 1.

From an ecosystem perspective, anthropogenic pressures result in the fragmentation of natural biomes, leaving a composite mix of different habitats. Remnant fragments that are representative of the original biome can be thought of as patches that exist within a matrix of habitats that are unlike the original 67, 68, 69. Interfaces between patches and the matrix exist at local-scales, and can be classified as **ecotones** – edges or transitionary zones between adjacent ecological systems where ‘biophysical factors, biological activity and ecological evolutionary processes are concentrated and intensified’ [70]. It has been suggested that by expanding ecotonal areas through interspersing human landscapes such as farmland and settlements with natural landscapes, anthropogenic influences can alter pathogen niches by bringing together humans, vectors, and reservoir hosts (wildlife or domestic animals), thus increasing contact and the risk of transmission [68]. Such landscape changes can be compounded by alterations in wildlife species interaction and abundance (e.g., host ecological traits); rodents can undergo ecological release at forest interfaces being attracted to farmland and human settlements for resources and suitable breeding habitat, and human settlements might provide suitable breeding habitat for mosquitos and birds (important arthropod vectors and reservoirs for West Nile virus) 70, 71. Evidence for an association between disease emergence and ecotones has been documented for several zoonoses with wildlife reservoirs, including yellow fever, Nipah virus encephalitis, influenza, rabies, hantavirus pulmonary syndrome, Lyme disease, cholera, *Escherichia coli* infection and African trypanosomiasis 70, 72, 73, 74. In urbanized areas such as cities, tangential variation in land use from rural–periurban–urban areas would be expected to generate a wide variety of ecotones on micro- and macrospatial scales. Ecotones can therefore represent important local-scale ecological interfaces within which zoonotic agents circulate and infect wildlife, domestic animals, and humans.

Another important factor in influencing interspecific wildlife contact and human–livestock–wildlife contact in urban environments is resource provisioning 19, 20. Clumping of resources occurs widely across urban environments at local (e.g., household) and landscape scales, whether as a result of variation in sanitation, refuse and agricultural byproducts, livestock-keeping practices, supplemental feeding of garden birds, or household food availability 13, 75, 76, 77, 78. Informal livestock keeping is commonplace in African cities, and often characterized by low biosecurity and mixed-species livestock being kept in close proximity to humans. Evidence from recent zoonotic emergence events in Asia (such as Nipah and highly pathogenic avian influenza viruses) and the circulation of relatively stable zoonoses (such as hepatitis E and bovine tuberculosis) implicate a role for livestock acting as bridge hosts, epidemiologically linking wildlife and humans 31, 79, 80. While resource provisioning commonly leads to increased contact rates between synanthropic wildlife, humans, and livestock, pathogen dynamics are also driven by susceptibility to infection, which, depending on the nature of provisioning, can be increased or decreased by host physical condition and immune defense 25, 78, 81. In Eastern Australia, the decline in natural food resources and abundance of flowering resources in urban gardens has resulted in increasingly large urban colonies of *Pteropus* spp. bats (flying foxes) existing sympatrically with human and horse populations. These bats act as a reservoir for Hendra virus, and have historically lived in widely dispersed, interconnected metapopulations. Plowright *et al.* [25] demonstrated that the effects of urban development on these metapopulations, through increased contact with humans and horses, and reduced connectivity between flying fox colonies, could dramatically influence the epidemic dynamics of the virus in flying foxes, and increase the risk of Hendra virus emergence in horses and people. Using mechanistic models, Becker and Hall [82] and Becker *et al.* [78] also demonstrated host demographic, contact and immunological effects of provisioning on R_0_, finding that unless provisioning reduces dietary exposure to pathogens or strongly improves host condition and immunity, increased aggregations of wildlife species dramatically increase pathogen invasion success and long-term prevalence. Environmental stressors such as heavy metal and pesticide pollutants, characteristic of certain urban environments, can further compound these outcomes through their effects on immunological function [83]. As such, resource provisioning is likely to increase host density (a key driver of transmission rates) and wildlife–livestock–human contact, making such areas important interfaces for disease emergence.

Table 1 applies the conceptual framework of wildlife–livestock–human interfaces developed by Jones *et al.* [8] to an urban setting such as Nairobi. Nairobi is a good example of a developing country city with human–livestock–synanthropic wildlife interfaces, and is a city, like many others, that has a growing boundary or edge which makes such contact more likely both on its edges and internally. In this context, we consider urban interfaces created through habitat fragmentation and resource provision. Such clear definition of interfaces is required to simplify the heterogeneous juxtaposition of humans and animals in urban landscapes, and thus enable the application of ecological, epidemiological, and anthropological approaches to the study of these landscapes. As well as capturing complex human and ecological processes that underlie disease emergence in urban landscapes, we believe that by studying these interfaces along rural –periurban–urban gradients, the landscape-level processes that accompany urbanization and underlie current theories of disease emergence could be captured.

## Concluding Remarks and Future Directions

In this review, we consider the role that urbanization plays in the emergence of zoonoses, through exploring the ecological complexity of wildlife–livestock–human interfaces. In doing so we argue that interfaces should be considered a critical component of disease ecology in changing urban landscapes, and echo a body of recent literature calling for greater ecological sophistication in epidemiological theories of disease emergence 84, 85, 86, 87. The majority of epidemiological studies use foundational concepts to study a single, or small number of well-characterized host species and pathogens when investigating transmission and connectivity within multihost systems. While this approach is well established, and useful in developing frameworks upon which the empirical characterization of a known host–pathogen system can be determined (through mechanistic models) and interventions planned (e.g., [34]), focus on a single species or pathogen might hinder the detection of pathogen emergence within a structurally complex system by overshadowing the evolutionary and transmission processes that precede this. As signaled by the emerging field of community disease ecology (reviewed in [87]), new approaches are required to investigate disease emergence, that shift focus from the pathogen to understanding the processes underlying emergence [35]. In response, disease ecologists have moved towards adopting principles from community ecology; including metapopulation and **network theory**, trait-based approaches and a consideration of processes acting across biological scales 27, 53, 84, 86, 87, 88, 89. The development of new modeling techniques will play a key role, and several frameworks have been suggested, that focus on integrating broad methodologies and crossdisciplinary collaborations to investigate causation in disease emergence 53, 90, 91. Such methods will be key to unraveling the structural complexity of ecological communities at wildlife–livestock–human interfaces, and thus understanding how they function as epidemiological systems prior to disease emergence.

While the focus of this review is on disease emergence, we would like to highlight the relevance of the frameworks discussed in combination with the broader concept of urban interfaces, for studying antimicrobial resistance (AMR). Currently considered urgent One Health issues, it is likely that the emergence of AMR and zoonotic pathogens in urban areas are underlined by a similar set of societal and ecological drivers [92]. Given the current rate of urbanization, and potential for associated changes in societal structure, food systems, and natural ecosystems to expose human and animal populations to novel pathogens, we recommend an interdisciplinary approach to studying urban human–wildlife–livestock interfaces, with the following aims: (i) establish characterizations for potential high-risk interfaces that exist along gradients of urbanization, and identify processes that have led to their formation; (ii) describe biological organization and community ecology at these interfaces, conduct surveillance for priority zoonotic pathogens (i.e., those with emergent potential) across host taxa, and study the evolutionary processes underlying cross-species transmission where it is detected (see Box 2); and (iii) at interfaces where transmission risks are identified, develop appropriate interventions that can be used to reduce risk of transmission. Given their epidemiological significance, interfaces represent a critical point of control for the transmission of zoonoses. A detailed discussion of control measures is beyond the scope of this article, but interventions could be implemented at an interface (i.e., preventative action such as husbandry and behavioral changes) or policy level (for a complete review, see [93]). If, as we discuss in this review, pathogen dynamics at interfaces are characterized by dynamic changes in community structure driven by abiotic factors, emphasis should be focused on studying epidemiological connectivity (i.e., pathways and heterogeneity of transmission – see Box 2) and how this changes longitudinally with time. Such studies will be crucial in identifying the dynamic processes responsible for driving changes in community structure and thus pathogen dynamics at different interfaces over time (see Outstanding Questions).

Outstanding Questions We consider the following unresolved questions as central to shedding light on the complex set of conditions required for a pathogen to enter a new host. Such studies will contribute to the development of more realistic mechanistic frameworks for cross-species spillover, and the design of appropriate interventions and control strategies.

**Characterization of interfaces**

At which urban animal–animal and animal–human interfaces is spillover of priority zoonotic pathogens most likely to occur?

What are the forces driving the creation of these interfaces?

What role does the environment and environmental change play in the transmission and spillover risk for zoonotic pathogens at these interfaces? How does this vary across gradients of urbanization?

**Interface dynamics**

*Reservoir communities and intermediate (bridge) hosts*

Wildlife reservoirs represent complex communities of maintenance and non-maintenance hosts, and conspecifics that could have a regulatory effect on parasite dynamics through ecological interactions with hosts. How are wildlife species assembled at proposed high-risk urban wildlife–livestock–human interfaces, and how does this vary across gradients of urbanization?

What is the presence and prevalence of zoonoses in urban synanthropic wildlife, and how does this vary across gradients of urbanization?

How are multispecies wildlife communities epidemiologically structured at high-risk urban wildlife–livestock–human interfaces, and how does this vary across gradients of urbanization?

What is the role of wildlife in contributing to genetic pools of antibiotic resistance across urban landscapes?

How does urban land-use change affect host fitness and immunity in synanthropic wildlife and livestock populations, and what effect (if any) does this have on circulating zoonotic pathogens?

How does microbial diversity in wildlife species (i.e., the pathogen pool) vary according to urban land-use change?

*Determinants of spillover*

Can pathogen sequence data shed light on adaptive and nonadaptive evolutionary processes occurring as pathogens are transmitted between species at urban interfaces? How do pathogen evolutionary processes relate to phylogenetic distance between reservoir, bridge, and target host species?

How does direct and indirect contact between wildlife, livestock and humans vary under differing livestock management conditions, and in response to broader biotic and abiotic factors in urban environments (e.g., anthropogenic behaviour, socioeconomic status, species diversity and climatic variation)?

What are the finer-scale epidemiological connections between synanthropic wildlife, livestock, humans, and their shared environments, and how is the risk of zoonotic pathogen transmission influenced by human and wildlife traits in urban environments (e.g., anthropogenic behavior, socioeconomic status, species diversity, and climatic variation)?

How does urban land-use change affect host fitness and immunity in synanthropic wildlife, livestock and human populations, and what effect (if any) does this have on circulating zoonotic pathogens?

## Figures and Tables

**Figure 2:**
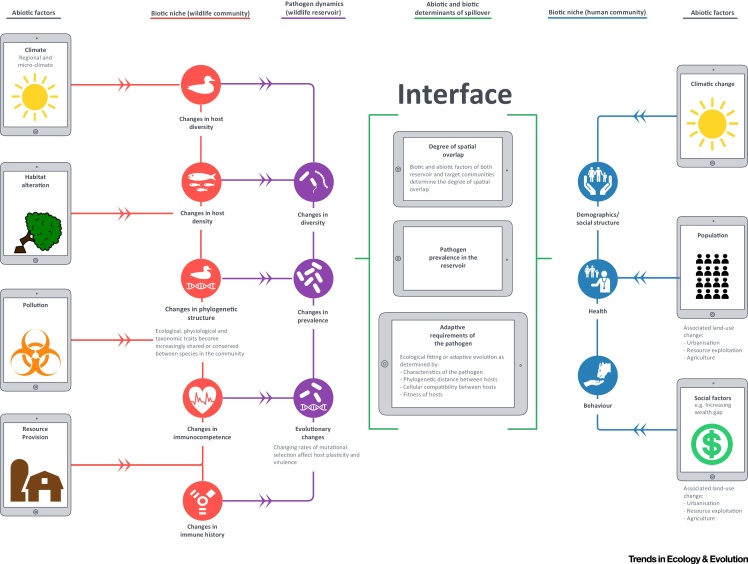
Cascades of Abiotic Factors, and the Components of Host–Pathogen Biotic System (for a Directly Transmitted Pathogen) That Are Affected by These Factors, Are Represented on Either Side of a Hypothetical Wildlife–Human Interface. Biotic systems represented here include a multihost wildlife community (acting as a parasite reservoir and composed of maintenance and non-maintenance hosts, and nonsusceptible species with direct ecological interactions with the reservoir), a human community (acting as variably susceptible target hosts) and the community of parasites within the wildlife reservoir. The requirements for spillover are represented centrally at the interface.

**Table 1:** A Framework for Wildlife–Human–Livestock Interfaces in a Developing City such as Nairobi^a^

Description	Examples	Proposed level of wildlife–livestock–human contact
Urban ecotonal interfaces and fragmentation of natural ecosystems (anthropogenically derived habitat edges)	Forest edge; agricultural edge; incursions for natural resource harvesting; urban wetlands	Increasing contact between humans, livestock and wildlife (both nonsynanthropic and synanthropic species)
Evolving urban landscape – areas of informally planned resource provision	Informal refuse dumps; increasingly intensive farming and associated value chains (low biosecurity); backyard farming	High contact between humans, livestock and synanthropic wildlife that is largely unmanaged
Managed urban landscape – areas of formally planned resource provision	Sewage plants; established intensive farming and associated value chains (high biosecurity)	Controlled contact between humans and livestock Little contact between wildlife, livestock, and humans
Managed urban landscape – areas of recreational habitat suitable for wildlife	Parks and recreation facilities; gardens	Few contacts between humans and livestock, and livestock and wildlife Increasing contact between humans and synanthropic wildlife

a Adapted from a broader conceptual framework describing types of wildlife–livestock–human interface and their characteristics, developed by Jones *et al.*[8].

## Box 1 Dynamics of Cross-Species Transmission at an Urban Interface

In considering how urbanization could drive the emergence of a directly transmitted zoonotic agent through changes to the distributional ecology of wildlife, it is helpful to consider urban land-use change as a combination of abiotic factors that can influence the biotic niche within which a focal host species and pathogen exist. In a classical ecological sense, abiotic niches are used to describe a set of conditions that permit persistence of a host within a certain geographical range (such as climate), while the host species biotic niche is characterized by the inhibitory or facilitatory impact of other species on its existence [54]. Biotic factors are likely to be scale dependent; ecological and epidemiological processes can operate differently at different scales. For example, the prevalence of rodent-associated zoonoses varies widely between and within cities, likely being determined by site-specific abiotic factors such as physical microenvironments [17]. Efforts to understand scale-dependent processes, such as the role of geographical distribution and ecological setting in creating opportunities for pathogen transmission to occur, have led to the emergence of landscape epidemiology – a discipline that incorporates the framework of traditional epidemiology with the field of landscape ecology to facilitate the study of pathogens in relation to the ecosystem in which it is found and the human population at risk [55]. This field would have application when addressing the complex and profound effects of urbanization on wildlife population dynamics, and thus the ecology of zoonotic agents in these systems.

At a hypothetical wildlife–livestock–human interface one can imagine a cascade of abiotic and biotic changes creating conditions suitable for disease emergence at different scales. Abiotic factors (which include climate, resource provisioning, pollution, and habitat alteration) exist at multiple scales and facilitate or inhibit the survival of new and existing wildlife species within the reservoir community, which dictates the structural assemblage and fitness of hosts (see Figure 2 in main text). At a finer scale, these factors influence the biotic niche and dynamics of pathogens within the system; abiotic changes can directly influence microbiota (e.g., driving antimicrobial resistance) 56, 57, whilst host diversity, density, phylogenetic structure (ecological, physiological and genetic similarity), and immunocompetence and immunological history of individuals all play an important role in host–pathogen interactions 58, 59. For example, not all conspecifics will be competent hosts for a given parasite, and as ‘dead-end’ hosts can play a role in regulating infection, while direct ecological interactions such as predation or competition will affect the population dynamics and distribution of competent reservoir hosts. Poulin [60] and Reperant [61] considered these factors as applied to the theory of island biogeography, where abiotic drivers influence the degree of interactions within source areas (sources of parasites such as wildlife reservoirs) and island areas (the recipient or target hosts), and the source-island distance (interactions between sources of parasites and recipient host populations that can drive spillover).

How these factors pertain to spillover to a target host (such as humans or livestock) is dependent on the force of infection; components of which include characteristics of the target host and the pathogen, and how the pathogen responds to changes in its biotic niche. Because abiotic factors influencing this system are driven by human activity, increased spatial overlap of humans, their livestock and wildlife is likely, but might not be enough to secure cross-species transmission. If the target host is human, then the likelihood of spillover can be moderated by individual and community variables such as social structure, living conditions, economic status, and health and risky behavior. From a pathogen genetic perspective, a jump into a new host species can either be nonadaptive (a process known as ecological fitting, where pre-existing traits allow emergence), or might require adaptive change (mutation of the pathogen in the reservoir host or the new host) [62]. Whether infecting new hosts is an adaptive or nonadaptive process depends on characteristics of the pathogen and host reservoir. Certain pathogens (RNA viruses in particular) seem inherently more adept at taking advantage of new epidemiological opportunities than others; possibly due to high mutation rates and broader host plasticity 52, 63. For others, structural properties of the reservoir can play a greater role and facilitate ecological fitting; phylogenetic distance between target (human) and reservoir hosts is a predictor of successful host jumps, while species belonging to certain phylogenetic clades might possess cellular components that make them more susceptible to pathogen invasion, regardless of phylogenetic distance from existing host species 52, 64. Benmayor *et al.* [65] showed that a higher density of susceptible hosts leads to an increased mutation rate and thus increased likelihood of viral host jumps occurring, while higher levels of interspecies transmission can lead to the adoption of more generalist pathogen virulence strategies [66]. Although conducted in unnatural microcosms, these studies demonstrate that the characteristics of the biotic niche of the pathogen, such as competition from sympatric microorganisms and host-reservoir dynamics, can also drive adaptive evolutionary processes. Finally, changes to population size and immune status of the reservoir can drive an increase in pathogen prevalence, thus amplifying the likelihood of spillover occurring. This leaves a key challenge to classify the levels of dynamic changes in organizational structure at different interfaces.

## Box 2 Future Perspective: Linking Community and Disease Ecology through Molecular Epidemiology

We consider how molecular epidemiology and network theory could provide a platform from which to investigate epidemiological connectivity, by mapping transmission and detecting pathogen adaptation across multiple scales of biological organization, and in doing so add real-world complexity to the study of disease emergence at interfaces.

**Studying Structural Complexity Using Networks**

By considering each species that exists within the community of interest as part of a network, it is possible to assess how species attributes (such as parasite diversity, preferred habitat, or social rank) vary within and between communities. In particular, the application of network theory to study epidemiology within ecological networks promises to address some of the shortcomings of approaches such as mathematical modeling and experimental studies (i.e., reductionist, lacking in biological reality), that are traditionally used to understand how pathogens behave in host communities. By capturing the structural complexity and heterogeneous mixing of individuals within a population, epidemiological networks can be used to investigate factors affecting transmission, while also providing a realistic framework for modeling pathogen spread through the community 94, 95, 96. However, the structure of epidemiological networks within multihost pathogen systems remains largely uninvestigated; the majority of studies that have applied network analysis to an epidemiological system for free-ranging wildlife consider transmission as a function of observed interactions between individuals belonging to a single species.

As initially demonstrated by VanderWaal *et al.* [97], molecular tools allow researchers to move beyond the assumption that observed contacts are reflective of transmission, by offering the potential to generate quantifiable measures of transmission between individuals of the same or different species. This has permitted studies to investigate community drivers of shared-parasite transmission; both VanderWaal and Atwill [98] and Blyton *et al.* [99] found covariance between networks of shared *Escherichia coli* genotypes and social contacts in giraffes and possums, respectively, such that individuals that were centrally located within social networks also acted as hubs of transmission. Gene dispersal measures represent just one **population genetics** approach to apportioning genetic variation that occurs within and between different populations, and have been successful to inform epidemiological connectivity between humans, wildlife, and livestock 72, 77. Others have used networks to describe population-level drivers in parasite diversity; Anthony *et al.* [100] developed network models of virus families in rhesus macaques, demonstrating that viral community assembly exhibits nonrandom patterns, which suggests that the effect of deterministic factors on viral diversity should be predictable. Thus, providing the limitations of a network approach are fully considered (reviewed in [94]), these tools could be used to describe the structure of wildlife communities implicated in zoonotic transmission to livestock and/or humans, identify key drivers that influence risk of transmission (such as land-use change or climate), and thus assist in untangling the complexity of epidemiological processes at interfaces in a realistic manner, regardless of the taxonomic distance between hosts.

**Phylogenetics**

Phylogenetic approaches focus on the study of evolutionary relationships among genetic lineages and can be used to reconstruct epidemiological histories from pathogen genetic sequence data. Given appropriate metadata (e.g., the date and host from which the sequence was sampled) it has been possible to reconstruct most likely populations of origin, historical host-switching events, and transmission pathways over long periods of time [101]. One potential approach for studying how ecology and spatial distribution of hosts affects the transmission and evolution of their parasites is to test for the association of host or environmental traits (such as species ecological characteristics, geographic location, behavior, or physical characteristics) with the phylogenetic structure of pathogen genetic sequences obtained from these hosts. For example, Parker *et al.* [102] developed a Bayesian Markov-Chain Monte Carlo approach for testing whether closely related taxa are more likely to share a trait of interest. This tool has proved useful in describing the spatial distribution of a number of pathogens (e.g., distribution patterns of yellow fever virus in Venezuela and evolutionary spread of influenza viruses in migratory birds 103, 104), and similar techniques have been embraced in community ecology [105]. Although the application of ecological trait-mapping onto phylogenies to understand how abiotic and biotic factors relate to parasite phylogenetic structure has not been explored for disease ecology, its potential is recognized by Suzán *et al.* [89].

By utilizing both population genetic (gene dispersal measures and network models) and phylogenetic trait-mapping approaches to investigate the epidemiological structure of multihost wildlife communities, it should be possible to depict epidemiological connectivity at an individual, intracommunity, and intercommunity scale. These techniques are therefore very appealing for determining connectivity within reservoir populations and between reservoir and target hosts at wildlife–livestock–human interfaces, and might provide an opportunity to inform the most appropriate targets for surveillance and control.

## Glossary

Basic reproductive number (R0): the expected number of secondary cases produced by a single infection in a completely susceptible population. In order for a pathogen to spread and be maintained within a population of animals, the value of R0 must be >1.
Bridge host: an epidemiologically functional host population within the disease reservoir framework, which is able to transmit a pathogen from the maintenance community to a target population. To occupy this role, a host must satisfy the following two prerequisites: (i) either be competent for infection, replication and excretion of the pathogen but unable to maintain it alone, or be capable of mechanical transmission; and (ii) occupy an ecological niche that facilitates direct or indirect transmission between maintenance and target hosts.
Ecotone: edges or transitionary zones between adjacent ecological systems where biophysical factors, biological activity and ecological evolutionary processes are concentrated and intensified.
Emerging infectious disease (EID): either a newly recognized, clinically distinct infectious disease, or a known infectious disease whose reported incidence is increasing in a given place or within a specific population.
Interface (disease): a boundary across which parasites can be passed between biological communities. For our use of this term, an interface is defined by the community of species on both sides of the boundary (i.e., human–livestock–wildlife), and the biotic niches within which these communities exist.
Land-use change: changes to the structure of ecosystems as a result of human activities, which lead to perturbation of biotic systems. Examples include: deforestation, expansion of agriculture, pollution, depletion of marine fisheries, and eutrophication.
Maintenance host/community: the populations making up a disease reservoir. Maintenance hosts are species within which a pathogen can persist without reintroduction from another host, while a maintenance community is composed of epidemiologically linked populations within which a pathogen can persist indefinitely.
Network theory: the theory underlying network models. At their simplest, these are an adjacency matrix consisting of nodes (vertices) that represent individuals within a population, and edges (links) that represent interactions between individuals. In an epidemiological context, this provides a framework for visualising potential pathways of transmission within populations.
Population genetics (epidemiology): the study of the distribution and change in frequency of alleles within or between populations, and how the influences of selection, genetic drift, mutation, and gene flow are scaled to an individual, group, population, and landscape level. In doing so, researchers can assess the consequences of microevolutionary processes at differing scales.
Phylogenetics: the study of evolutionary relationships between individuals or species. These relationships are represented as a phylogeny (or evolutionary tree), consisting of a set of nodes (branching points) and edges.
Reservoir of infection: one or more epidemiologically connected populations in which a pathogen can be permanently maintained and from which infection is transmitted to a target population (such as humans).
Spillover: the disease dynamics that enable a pathogen to be transmitted into a susceptible target host population from its reservoir population.
Synanthropic wildlife: wildlife species that are ecologically associated with humans.
Target host: an epidemiologically functional host population within the disease reservoir framework, which is the focus for disease control.
